# Two-photon fluorescence imaging of subsurface tissue structures with volume holographic microscopy

**DOI:** 10.1117/1.JBO.25.12.123705

**Published:** 2020-11-23

**Authors:** Xiaomin Zhai, Sunil Vyas, J. Andrew Yeh, Yuan Luo

**Affiliations:** a National Taiwan University, Institute of Medical Device and Imaging, Taipei, Taiwan; b National Tsing Hua University, Department of Power Mechanical Engineering, Hsinchu, Taiwan; c National Taiwan University, Molecular Imaging Center, Taipei, Taiwan; d National Taiwan University, YongLin Institute of Health, Taipei, Taiwan

**Keywords:** two photon microscopy, volume gratings, holography, imaging systems, microscopy, fluorescence microscopy

## Abstract

**Significance:** Two-photon (2P) fluorescence imaging can provide background-free high-contrast images from the scattering tissues. However, obtaining a multiplane image is not straightforward. We present a two-photon volume holographic imaging (2P-VHI) system for multiplane imaging.

**Aim:** Our goal was to design and implement a 2P-VHI system that can provide the high-contrast optically sectioned images at multiple planes.

**Approach:** A 2P-VHI system is presented that incorporates angularly multiplexed volume holographic gratings and a femtosecond laser source for fluorescence excitation for multiplane imaging. A volume hologram with multiplexed gratings provides multifocal observation, whereas nonlinear excitation using a femtosecond laser helps in significantly enhancing both depth resolution and contrast of images.

**Results:** Standard fluorescent beads are used to demonstrate the imaging performance of the 2P-VHI system. Two-depth resolved optical-sectioning images of fluorescently labeled thick mice intestine samples were obtained. In addition, the optical sectioning capability of our system is measured and compared with that of a conventional VHI system.

**Conclusions:** Results demonstrated that 2P excitation in VHI systems provided the optical sectioning ability that helps in reducing background noise in the images. Integration of nonlinear fluorescence excitation in the VHI provides some unique advantages to the system and has potential to design multidepth optical sectioned spatial–spectral imaging systems.

## 1 Introduction

Light-induced fluorescence imaging has widely been used for biomedical and clinical applications.^1^^,^^2^ Two-photon (2P) microscopy is a well-established technique and preferred for biomedical imaging.^3^ Two-photon excitation is a nonlinear fluorescence process in which the simultaneous absorption of two photons occurs.^4^^–^^6^ Typically, a 2P excitation in microscopy is achieved using an intense ultrashort infrared light source. It provides deep penetration into the tissue in comparison to confocal or conventional fluorescence microscopy. One of the main advantages of a 2P microscope is its ability to maintain high contrast within the thick samples, which helps in studying biological structures deep within the tissues. The longer excitation wavelength (twice the wavelength with single photon) scatters less and allows deep penetration into biological samples. In addition, wide spectrum separation between the excitation and emission wavelengths results in the rejection of the background scattered light with minimum signal loss. Fluorescence in a 2P microscope occurs only at the focal volume where the density of the photon is very high, which results in optical sectioning ability and depth discrimination.

A variety of three-dimensional optical imaging systems based on volume holography have recently been developed.^7^ In general, a volume holographic imaging (VHI) system consists of a 4-f imaging system with volume holographic gratings at the Fourier plane to simultaneously observe different depth images within a specimen. VHI systems offer multiple advantages over conventional imaging systems. They have been implemented with widefield as well as point scanning microscopy systems to obtain 3D fluorescence signals from biological samples.^2^^,^^7^^–^^10^ The shift-variant point spread function of a volume holographic grating in imaging provides a unique spatial–spectral feature that allows discrimination of spatial and spectral information from multiple planes. This technique has been used for fluorescence and nonfluorescence imaging under different modalities. These include multiplane microscopy, hyperspectral imaging, and wavelength-depth selective microscopy.^7^ The purpose of implementing multiplexed volume holographic grating in imaging systems is to parallelize the imaging process by reducing the scanning time. The integration of the angularly multiplexed volume holographic gratings (AMVHGs) in imaging allows the simultaneous observation of multiplane images from the focal region. Recently, it has been shown that simultaneous multiplane images of up to eight different planes can be obtained in a single measurement.^7^ However, VHI typically uses single-photon excitation and provides poor optical sectioning to discriminate in-focus fluorescence signals and out-of-focus backgrounds, which limits prior VHI system work for tissue imaging. As is well known in microscopy, 2P excitation techniques^11^^–^^14^ are superior to single-photon excitation microscopy due to virtual biopsy with greater tissue penetration depth and less photobleaching.^11^ In a 2P microscope, due to nonlinear dependence of emission intensity on the excitation intensity, fluorescence emission occurs only from the focal region where the density of the photon is very high and the system naturally acquires optical sectioning ability and depth discrimination. To the best of our knowledge, no VHI system combined with two-photon excitation has been developed for tissue imaging. In this work, we demonstrate a proof-of-principle microscopic imaging system that incorporates angle multiplexed volume holographic gratings and a femtosecond laser to observe multidepth resolved two-photon fluorescence images of biological samples. We experimentally measured the optical sectioning capability of our system and acquired femtosecond laser-induced fluorescence images of mouse intestine samples with fine depth resolution.

## 2 Methods

### 2.1 Angularly Multiplexed Volume Holographic Gratings

We designed and fabricated a volume phase grating recorded onto a photopolymer. The quality of the reconstructed field generated from the photopolymer-based volume hologram is mostly determined by the quality of the photosensitive material. Phenanthrenquinone (PQ)-doped polymethyl methacrylate (PMMA) polymer material (PQ:PMMA) has been regularly used for designing multiplexed volume gratings due to its excellent optical properties, such as high optical sensitivity, diffraction efficiency, and stability with negligible shrinkage.^2^ It can provide a large refractive index modulation for recording high spatial frequencies. To fabricate a volume holographic grating, an interference pattern between the signal and reference wave is stored as the permanent changes in the refractive index of the medium that embed all the amplitude and phase information of the recorded beams. The refractive index modulation inside the medium is given by

$$n=n_{0}+n_{1}\;\cos({\overset{\to }{K}}_{G}.\overset{\to }{r}).$$ (1)

A multiplexed hologram is recorded in a photopolymer substrate by the interference of the signal and reference beam according to Eq. (1). The grating vector of each gratings in AMVHG is given by ${\vec{K}}_{Gi}$^15^

$${\overset{\to }{K}}_{Gi}={\overset{\to }{K}}_{Ri}-{\overset{\to }{K}}_{Si},\;i=1,2,$$ (2)

K→Ri=k sin θRix^+k cos θRiz^, (3)

K→Si=k sin θSix^+k cos θSiz^, (4)

where $\theta_{Ri}$, and $\theta_{Si}$ are the incident angle of reference and signal beams, respectively, and k=2π/λ, where λ is the operation wavelength. Following the previous method, the recording angles for the first grating are ($\theta_{R1}=30\;\deg$ and $\theta s_{1}=-30\;\deg$).^15^ Keeping the identical signal beam angle as the first grating, the reference beam angle for recording the second gratings is $\theta_{R2}=32\;\deg$. The PQ:PMMA hologram substrate was ∼1.8-mm-thick and both gratings were recorded using an argon ion laser operating at a wavelength of 488 nm and designed to probe green fluorescence images.^4^^,^^6^ The Bragg wavelength degeneracy^16^^–^^19^ between recording and probing is given by

$$\Delta \theta =\theta_{Ri}-\phi +\cos^{-1}(\lambda_{0}+\Delta \lambda /2\Lambda n).$$ (5)

**Fig. 1:**
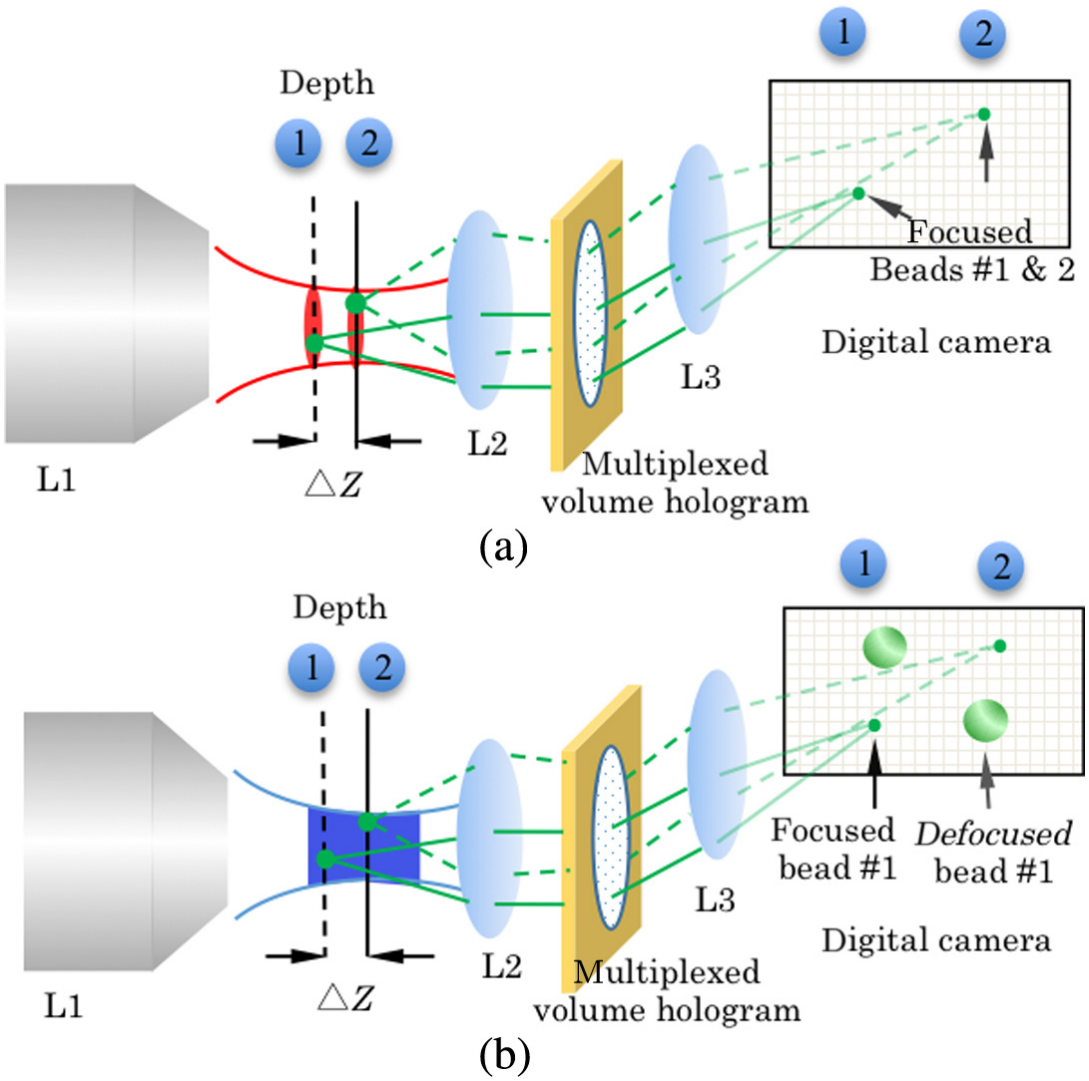
Schematic drawing: (a) the proposed 2P-VHI system and (b) conventional VHI system using single-photon excitation. Defocused beads images, shown in (b), are much suppressed in (a) due to two-photon excitation.

In the imaging system, an AMVHG, under a Bragg-match condition,^3^^,^^16^^,^^17^ acts like a multifocus lens and simultaneously displays laterally separated multidepth images of volumetric samples onto the CCD as schematically shown in Fig. 1. The grating period of the volume phase grating recorded in substrate is given by

$$\Lambda =\frac{1}{2n\;\cos(\phi -\theta_{Ri})},$$

where Δθ is the angular shift of the probe beam with operation wavelength ($\lambda_{o}+\Delta \lambda =532\;\mathrm{nm}$) from the Bragg-matched angle $\theta_{Ri}$ of the recording beam at $\lambda_{o}=488\;\mathrm{nm}$. ϕ represents the slant angle of the grating vectors and n is the index value of the hologram substrate. In our experimental n∼1.49, the first multiplexed grating is unslanted (ϕ=90  deg), whereas the second multiplexed grating is slanted. The interference pattern between a reference and the signal wave was recorded in PQ:PMMA substrate to create a VHG, with an exposure time of 25 s and laser power of ∼0.4  W.

### 2.2 Two-Photon-VHI System

We built the 2P-VHI system as shown in Fig. 1. It has two main parts, a two-photon illumination system and a VHI system incorporating AMVHGs.

2P illumination of the sample is obtained using a tunable mode-locked Ti:sapphire laser (800 nm, Spectra-Physics) and an Olympus microscope objective lens L1 (MSPlan100×, NA=0.95). The imaging part of the microscope consists of Olympus objective L2 lens (MSPlan50X), and a specially designed AMVHG, a Mitutoyo L3 lens (MPlanAPO10X), to obtain a 4f imaging system as shown in Fig. 1. The output from the Ti:sapphire laser was coupled into the microscope and focused to provide a beam waist close to the back aperture of the objective lens. This laser delivered a time-averaged power of 1.7 W and a repetition rate of 100 MHz with a pulse duration of 100 fs. The power of the laser was attenuated using a variable neutral density filter. All the images were recorded with a high-resolution Andor camera (iXon 897). In this study, the volume hologram consists of two AMVHGs and is positioned at the Fourier plane. When the Bragg-matched condition is satisfied, then gratings act as a multifocus lens and display laterally separated multidepth images of volumetric samples onto the CCD as shown in Fig. 1. The depth separation (Δz) of the two multiplexed gratings was ∼35  μm to probe two different focal planes. The depth separation can be tuned while recording the multiplexed grating.

## 3 Results

The difference between the conventional VHI system that uses one-photon fluorescence excitation and the 2P-VHI system is depicted in Figs. 1(a) and 1(b), respectively. In the 2P-VHI system, signal from the two depths separated by a distance (Δz) can be resolved due to the inherent optical sectioning capability acquired by the system due to the improved point spread function in the axial direction.^4^ As shown in Fig. 1(a), the two red focal spots corresponding to the two different depth illuminations can be obtained by scanning the illumination objective in the axial direction. The ability of AMVHGs to acquire multifocal depth images is evident in this situation. The high angular selectivity obtained from the Bragg-matched condition provides the depth selectivity in the imaging part of the microscope. It is important to note that with the conventional VHI system using single-photon excitation, this depth discrimination is obscured with the out of focus background light as shown in Fig. 1(b). The use of the two-photon fluorescence excitation alleviates this problem in the conational VHI system and shows better imaging performance.

### 3.1 Imaging of Fluorescence Beads

**Fig. 2:**
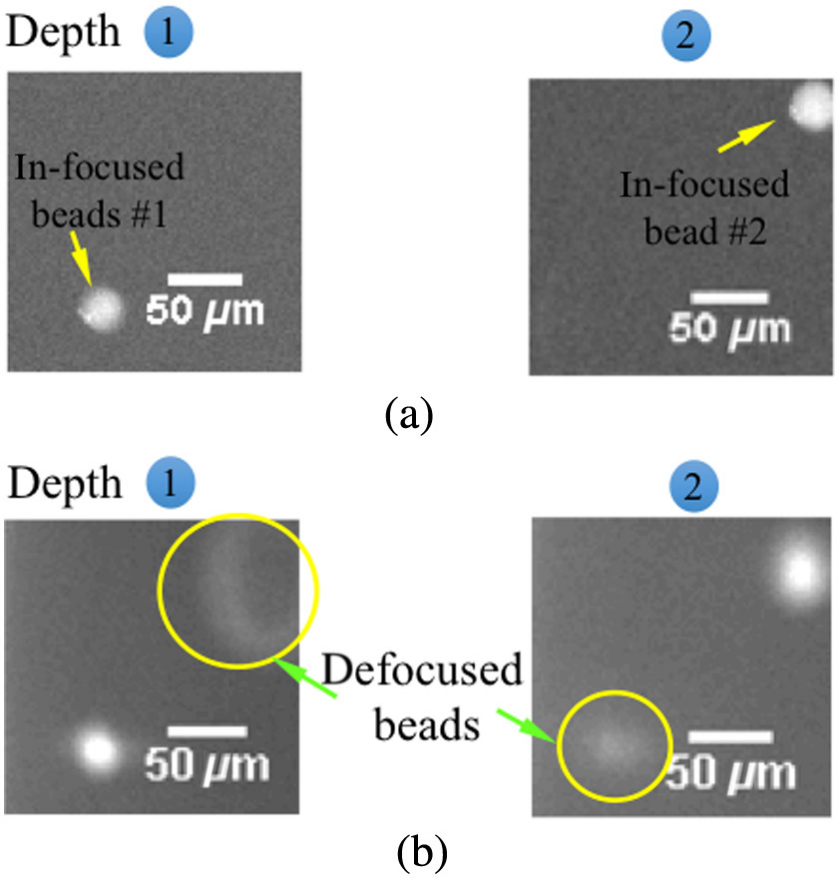
(a) Images of the fluorescently labeled microspheres from 2P-VHI system. Only optical sectioned images with in-focus signals are observed from the CCD. The two depth images were observed by scanning the illumination in the axial direction. (b) Conventional VHI system with single-photon excitation obtained with LED illumination. Images of 15-μm fluorescence microspheres for the two axial planes. Both out-of-focus and in-focus microspheres are captured from the CCD. Note that the two images were captured without axial focus scanning adjustment.

The ability of the proposed 2P-VHI system to resolve multiple depths in volumetric samples was verified by imaging fluorescence-labeled microspheres (15-μm-diameter, Polysciences) suspended within a thick slab of agarose (Invitrogen). Figure 2 provides comparisons of standard conventional VHI systems with single-photon excitation and the optically sectioned images captured by 2P-VHI systems, respectively. Two depth-resolved images with defocused light at both planes observed from the CCD with quasi-collimated LED illumination are shown in Fig. 2(a). Figure 2(b) shows the resultant images from the 2P-VHI system, and out-of-focus background noise is rejected from the desired in-focus signal.

### 3.2 Measurement of Optical Sectioning Capability

**Fig. 3:**
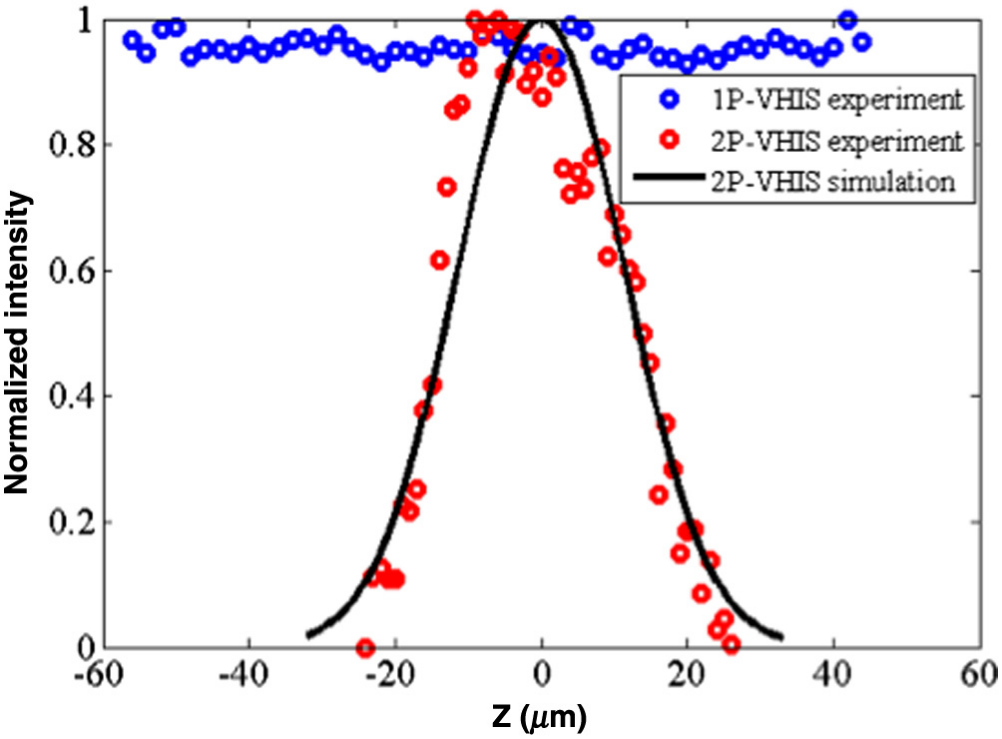
Comparison of experimentally measured depth selectivity between conventional VHI system (blue dots) and 2P-VHI systems (red dots) with curve fitting in solid line.

To further investigate the optical sectioning capability of our 2P-VHI system, we experimentally measured the depth selectivity of the system. A thin sample of diluted fluorescein solution was evenly spread between cover slits.^14^ The thickness of the fluorescein solution was ∼1  μm. The thin fluorescence sample was moved by controlling a motorized piezo-stage (MAX341, Thorlabs) at a 0.8-μm step size along the axial direction, and defocused images were captured on the camera. In Fig. 3, red dots show the normalized total intensity of each defocused plane image versus the defocus distance for the 2P-VHI system, whereas the blue dots represent normalized total intensity for a conventional wide-field VHI system illuminated with a quasi-collimated LED source. Comparison of depth selectivity to the conventional VHI system with 2P-VHI system is shown in Fig. 3 and the measured full-width at half-maximum (FWHM) of the 2P-VHI system is 24  μm. The conventional VHI system shows no depth selectivity. A drastic change in the depth selective property of the VHI system can be obtained by changing the illumination from one photon with the 2P illumination. Together with all the inherent advantages of the spatial–spectral imaging, the VHI system acquires fine optical sectioning ability, which further enhances its performance for high-contrast 3D imaging for various applications.

Depth resolution will largely depend on the NA of the lens and the wavelength. In our experiment, we used an MSPlan 100× objective lens with an NA of 0.95. At present, we used two angularly multiplexed grating with the recording parameter described previously. The separation between the two angularly multiplexed grating depths can be altered during the recording process.

### 3.3 Multiplane Imaging of Bio-Samples Using 2P-VHI System

**Fig. 4:**
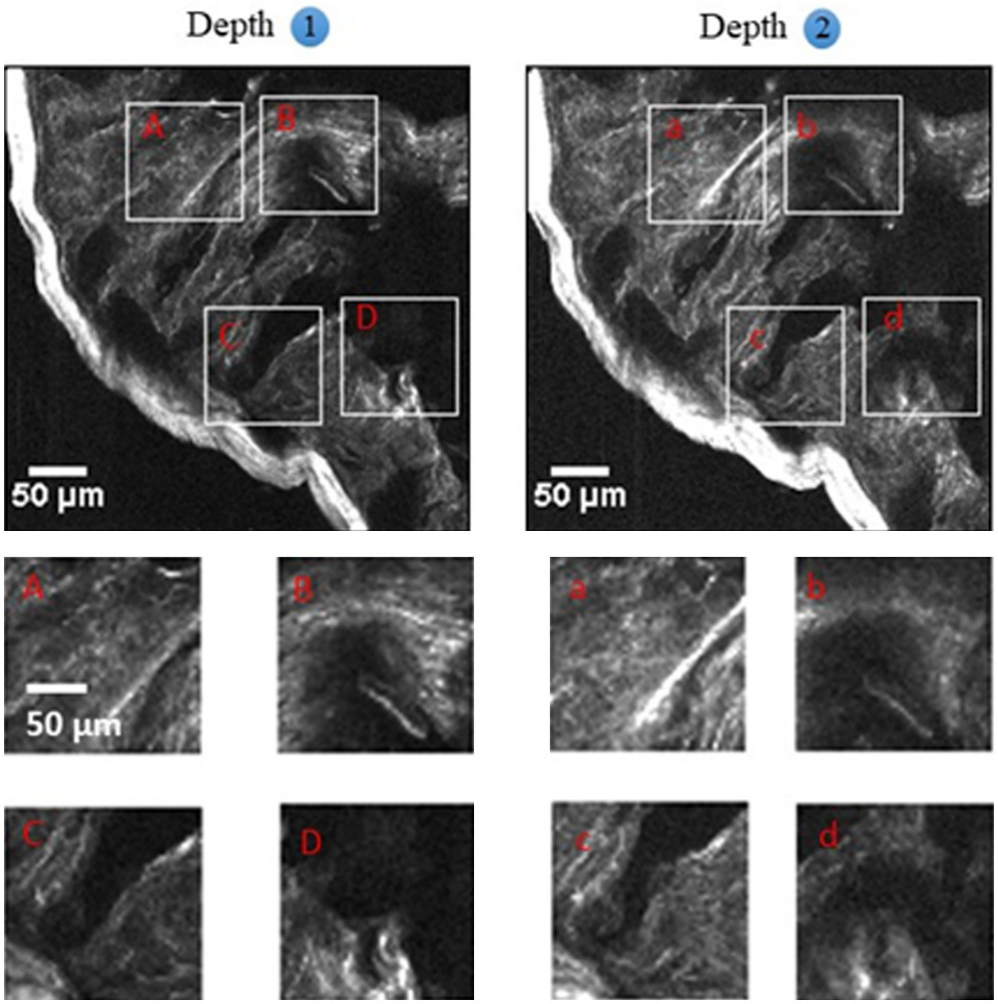
Two-depth resolved optical-sectioning images of fluorescently labeled mice intestine sample obtained using the 2P-VHI system. (A)–(D) Detailed resultant images at selected locations of the intestine sample in depth 1. (a)–(d) Detailed images of the sample in depth 2. Two planes are separated by 35  μm.

Experiments using fluorescently labeled microspheres are a condition where the sample is sparse. However, practical imaging for biomedical applications, in reality, often requires highly scattering samples. To test the performance of the 2P-VHI system for imaging dense and scattered samples, we used intestinal tissues of mice. The intestines were fixed in 4% paraformaldehyde overnight at 4°C. Subsequently, the sample was permeabilized by being incubated in 0.1% Triton-X 100 following which the intestine sample was stained with Alexa-488 labeled Phalloidin for green fluorescence labeling of actin proteins. The sample was washed thoroughly, placed between two coverslips in phosphate buffered saline medium, gently compressed, and sealed. The thick sample was then imaged with the 2P-VHI system. Figure 4 shows the images of the villi of the mouse intestine sample as taken with our 2P-VHI system. Two planes, separated by 35  μm, are imaged onto different lateral parts of the camera. Figures 4(A)–4(D) show the resultant images at depth 1, whereas Figs. 4(a)–4(d) show images at depth 2. These images clearly show that we can achieve good out-of-focus background rejection of multiple fluorescent planes using a 2P-VHI system for biological samples. We show a proof of principle experiment by presenting two depth discrimination images by our system. By increasing the number of layers in the AMVHGs, we can acquire multiple depth images with high contrast even for high scattering tissues. Our system provides the advantage of inherent VHI and the fine high-contrast optical sectioning of 2P excitation.

## 4 Conclusions

In summary, a 2P-VHI system was designed and implemented to observe multiple depth-resolved images of tissue samples with fine optical sectioning. The addition of 2P sources in the volume holographic system improves the optical section performance of the system, which resulted in high-contrast images. Our system is not limited to the 2P excitation and it can readily be adapted for multi-photon excitation microscopy. The system can be extended to provide more focal planes by designing high-dimensional multiplexed gratings. The inherent wavelength degeneracy offered by volume grating can be used to observe multicolor fluorescence images.
